# Multimodal Imaging in Unusual Alport Retinopathy

**DOI:** 10.7759/cureus.52768

**Published:** 2024-01-23

**Authors:** William Evans, James Richardson-May, Rashi Arora

**Affiliations:** 1 Ophthalmology, Salisbury District Hospital, Salisbury, GBR; 2 Ophthalmology, Salisbury District Hospital, Southampton, GBR

**Keywords:** alport retinopathy, fundus photo, autofluorescence, oct, multimodal imaging, alport syndrome

## Abstract

Alport syndrome, a rare genetic condition, can manifest various ocular abnormalities. This case report presents a unique instance of Alport syndrome where bilateral reduced visual acuity led to cataract surgery and subsequent central serous chorioretinopathy due to steroid treatment. By utilizing multiple imaging modalities, we aim to illustrate classical and atypical findings, addressing a literature gap and sharing our experience for educational purposes.

## Introduction

Alport syndrome is a rare genetic condition characterised by mutations in collagen IV genes, resulting in structural abnormalities in collagen IV chains. This leads to a multisystem disease affecting glomerular, cochlear, and ocular basement membranes, posing a heightened risk of kidney failure, sensorineural hearing loss, and ophthalmic abnormalities [1,2].

Ocular complications in Alport syndrome commonly include dot-and-fleck retinopathy, characterised by white or yellow granulations around the macula [3,4]. Patients may also exhibit anterior lenticonus, in which the lens surface protrudes through a thinned capsule [3,5]. Thinning of the retina is also prevalent and may predispose to symptoms and retinopathy in more extensive disease [3]. Severe thinning of the membrane can cause dots and flecks to demarcate from the fovea, resulting in a dull macular reflex or “lozenge”, another characteristic feature of the disease [4].

A comprehensive literature review on ocular manifestations of Alport syndrome revealed many examples of the typical features of the disease [6]. While some examples of characteristic imaging were found, these were limited and remained challenging to visualise [4,7,8]. However, there was a scarcity of reports detailing cases of the condition alongside comprehensive multimodal imaging to elucidate findings in more detail.

We describe a patient with Alport syndrome who presented with gradual vision loss, underwent bilateral cataract surgery, and developed central serous chorioretinopathy (CSCR) as a result of topical steroid treatment. We aim to demonstrate the photos, autofluorescence and optical coherence tomography (OCT) images typical in these patients.

We found no reports of patients with Alport syndrome who developed central serous chorioretinopathy as a response to steroids, though steroid use is well known to be a common association of CSCR.

## Case presentation

A 47-year-old male presented to the ophthalmology department with a gradual, progressive, bilateral decline in vision and nyctalopia over several months. He has a medical history of X-linked Alport syndrome, with a pre-existing amblyopic left eye. Notably, he had previously undergone a renal transplant and requires hearing aids, both as a result of Alport syndrome-related damage.

Upon examination, subtle corneal basement membrane changes were observed in both Descemet’s and Bowman’s layers with corresponding guttate and signs of epithelial basement membrane dystrophy, consistent with posterior polymorphous dystrophy. The patient was emmetropic and axial length was 23.31 in the right eye and 23.37 in the left eye. Symmetrical posterior lens changes were also identified, believed to be bilateral polar and posterior subcapsular cataracts.

Fundus examination revealed distinctive characteristics, including a poor macular reflex, dot-and-fleck retinopathy and areas of peripheral retinal pigmentation.

Additionally, the pattern electroretinogram exhibited marginal degradation and broadening while the small check visual evoked potential was significantly degraded. All else was normal. Both findings are consistent with reduced macular function, attributed to either the presence of cataracts or macular dysfunction.

The patient acknowledged a guarded prognosis but consented to bilateral phacoemulsification and intraocular lens implantation, conducted on separate occasions without complications, and starting postoperative topical 1% dexamethasone, four times a day for four weeks.

While there was a significant improvement in visual acuity for the patient, a subsequent postoperative OCT done at the four-week follow-up revealed the emergence of new subretinal fluid in each eye, coinciding with the commencement of dexamethasone therapy.

Multimodal imaging

**Figure 1:**
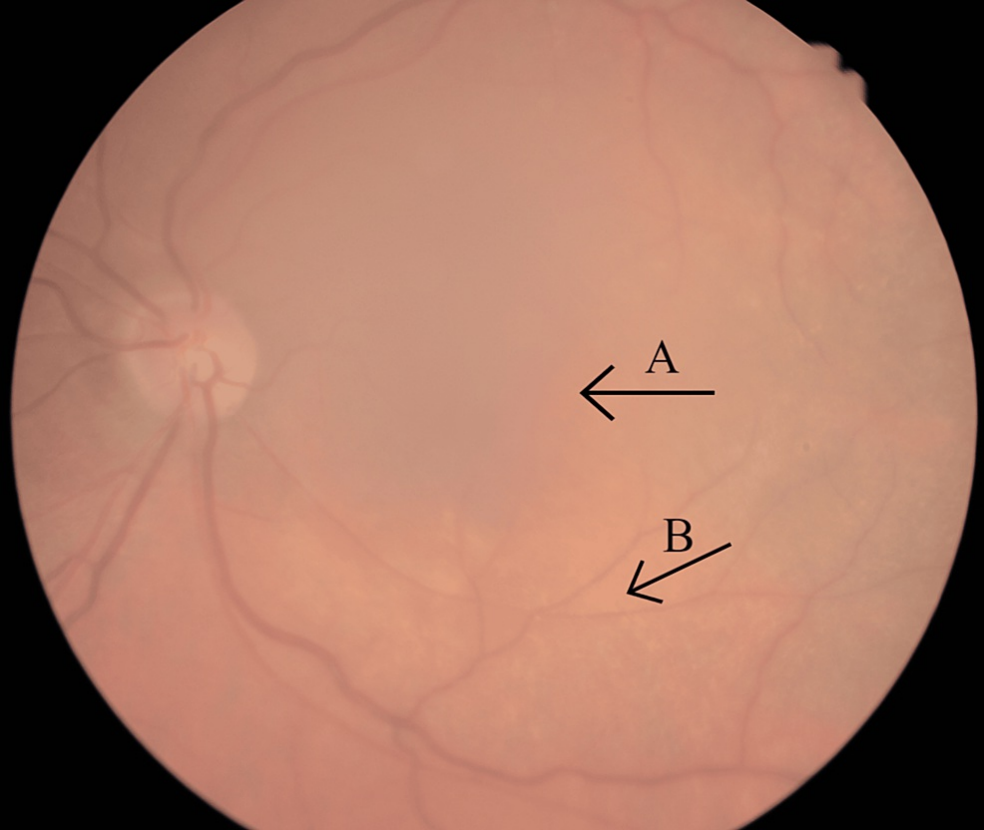
Left eye fundus photograph Arrow A indicate the "lozenge", or dull macular reflex, whereas arrow B indicates the scattered dot-and-fleck retinopathy. The presence of the cataract obscures the image.

**Figure 2:**
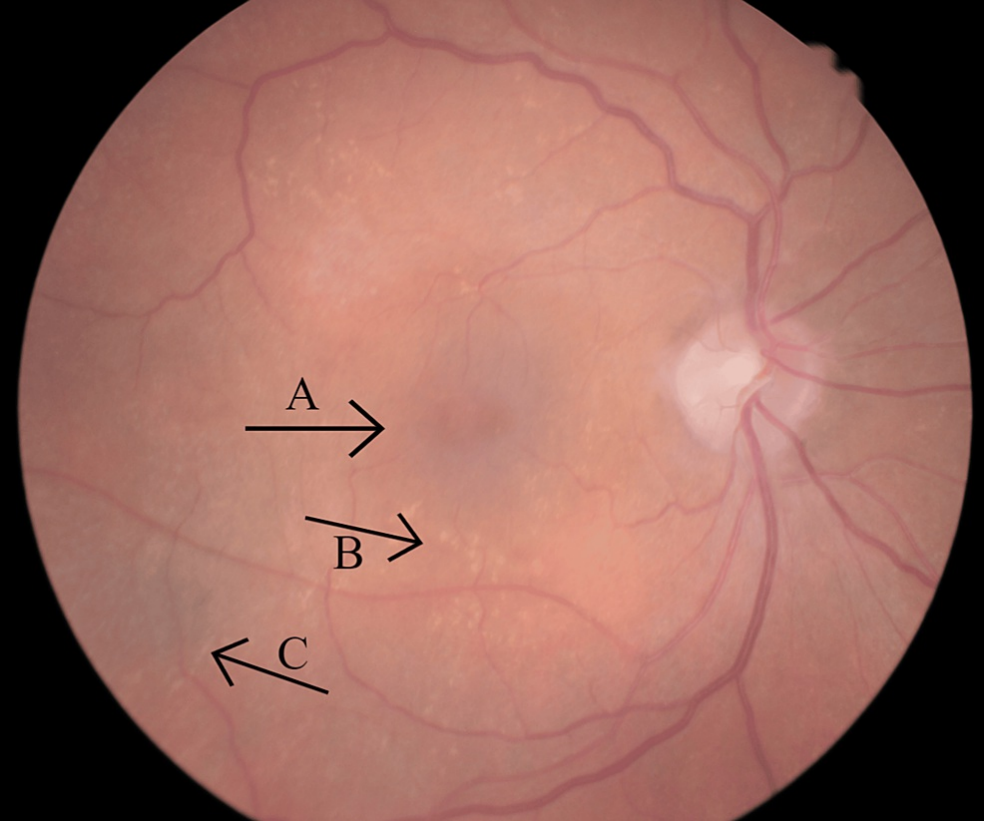
Right eye fundus photograph Arrow A indicates the "lozenge" or dull macular reflex. Arrow B highlights the scattered dot-and-fleck retinopathy characteristic of Alport retinopathy. Also visualised is peripheral pigmentation, marked via arrow C.

Preoperative fundus photos of each eye illustrate the 'lozenge' characteristic of severe forms of the disease, as presented in Figures 1, 2. Notably, areas of peripheral retinal pigmentation in the right eye, less typical of Alport syndrome, are visible. Additionally, the photographs show the distinctive dot-and-fleck retinopathy surrounding the macula, with a more pronounced manifestation in the right eye.

**Figure 3:**
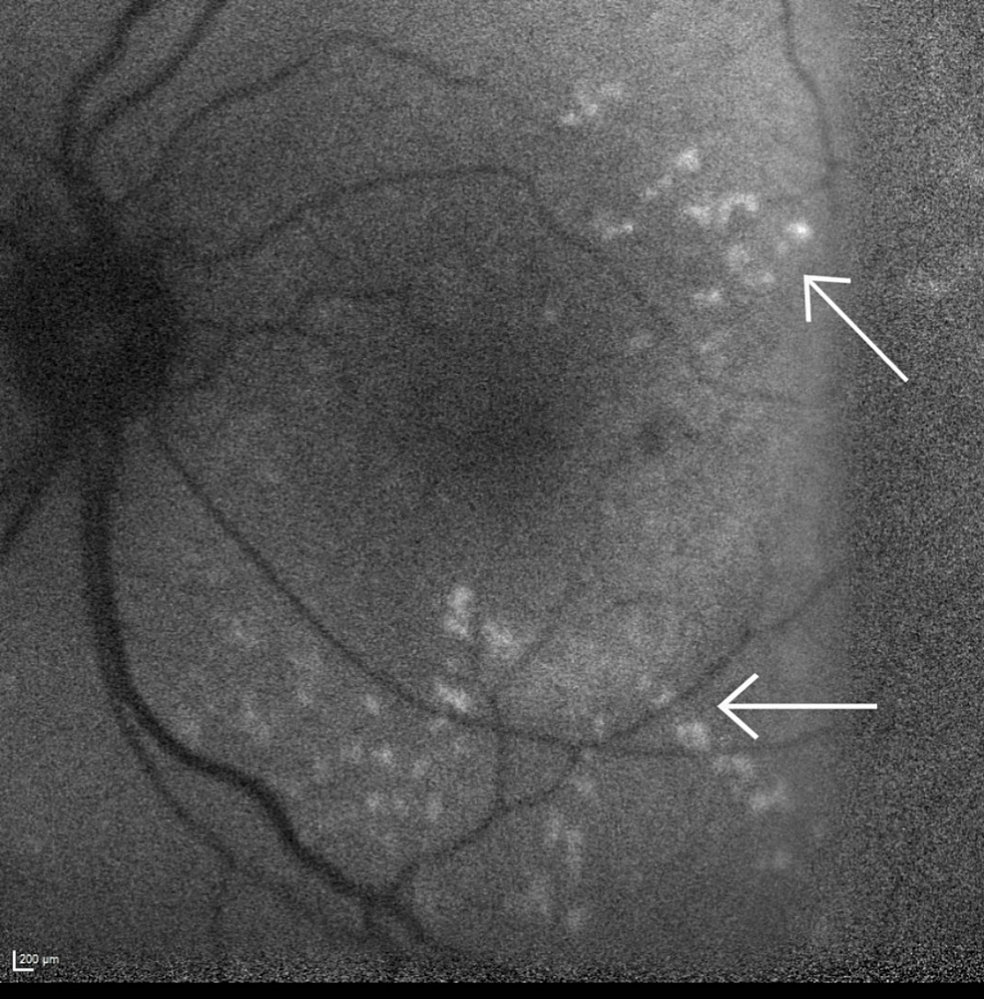
Left eye autofluorescence Both arrows highlight the characteristic dot-and-fleck retinopathy seen in Alport syndrome as multiple speckled areas of hyper-autofluorescence scattered in the posterior pole.

**Figure 4:**
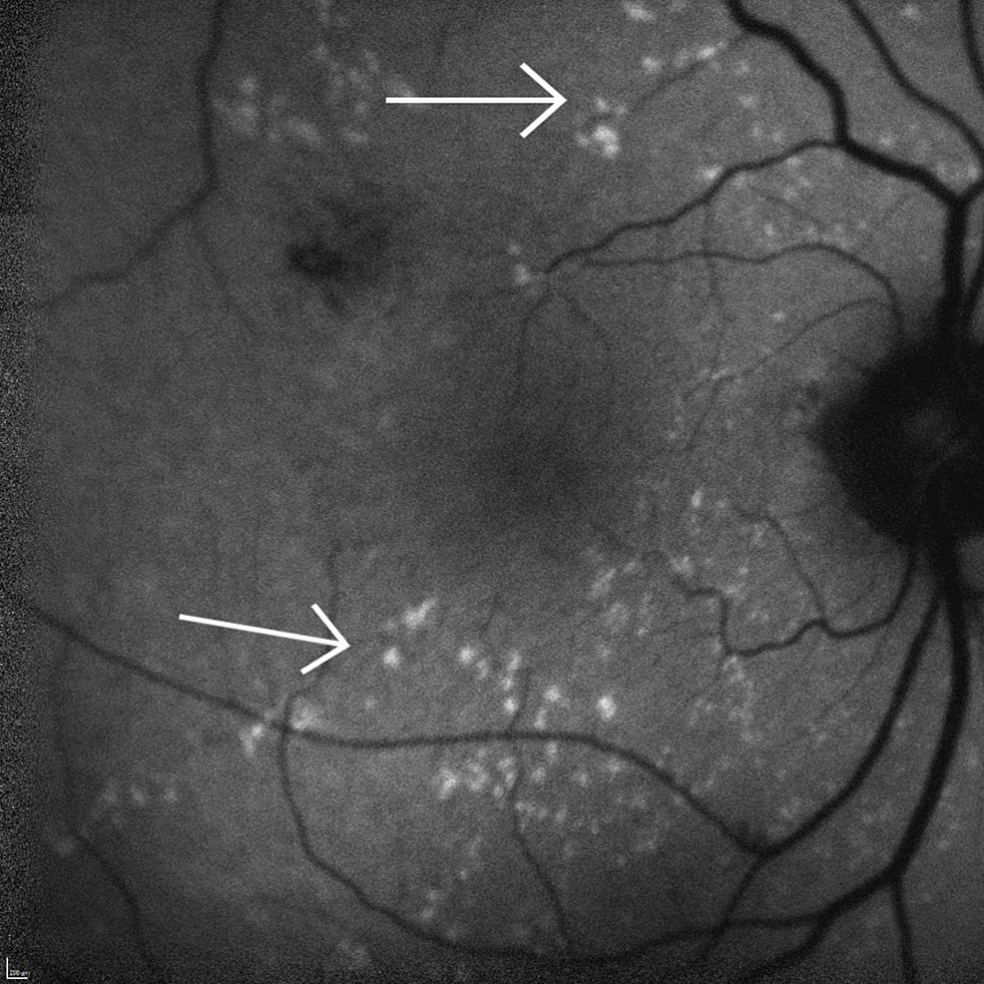
Right eye autofluorescence Both arrows highlight the characteristic dot-and-fleck retinopathy seen in Alport syndrome as multiple speckled areas of hyper-autofluorescence scattered in the posterior pole.

A more detailed examination is provided through autofluorescence images (Figures 3, 4), clearly depicting the dot-and-fleck retinopathy. These images reveal multiple speckled areas of hyper-autofluorescence scattered in the posterior pole, with a more prominent distribution along the superior and inferior arcades.

**Figure 5:**
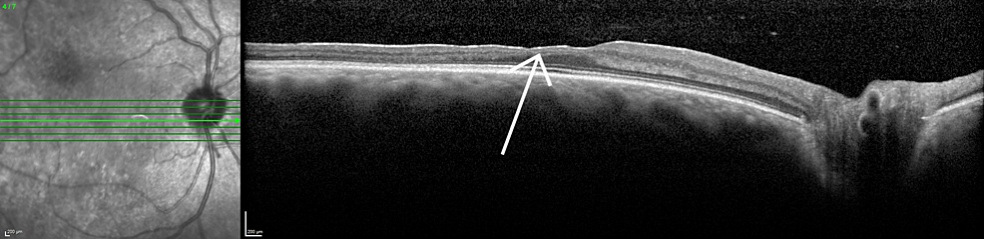
Optical coherence tomography of the right eye (foveal) The arrow indicates areas of retinal thinning.

**Figure 6:**
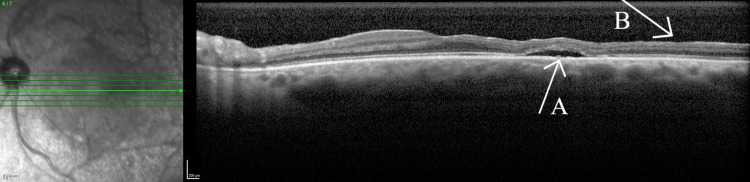
Optical coherence tomography of the left eye (foveal) Arrow A illustrates the subretinal fluid developed postoperatively. Arrow B further illustrates areas of retinal thinning.

**Figure 7:**
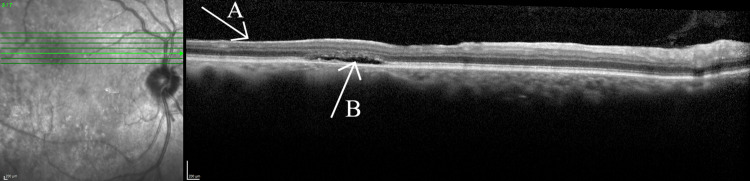
Optical coherence tomography of the right eye Arrow A highlights retinal thinning. Arrow B highlights the subretinal fluid the patient developed postoperatively.

Preoperative OCT scans revealed areas of retinal thinning throughout the entire posterior pole. Areas of thinning for the right eye are shown in Figure 5. Postoperatively, Figures 6, 7 illustrate subretinal fluid that the patient has developed, along with patchy hyperreflective lesions along the roof of the fluid cavity, in keeping with CSCR.

Differential diagnoses and case outcome

Despite the patient already having a known diagnosis of Alport syndrome, other causes for the imaging findings must be considered.

The dot-and-fleck retinopathy, although characteristic of Alport syndrome, may be difficult to distinguish between retinal flecks also seen in many other inherited disorders, such as fundus albipunctatus, fleck retina of Kandori or familial drusen. In addition, with the patient presenting nytalopic, other retinal dystrophies must be considered; the most common of these being retinitis pigmentosa. These other pigmentary retinopathies typically remain without macular involvement, however, and are not associated with the pattern of systemic disease this patient has a background of [9,10]. In addition, his known genetic condition and other findings on imaging make these unlikely. Thus, the imaging findings were attributed to Alport retinopathy, with the presenting symptoms being due to co-existing bilateral polar cataracts.

These findings can be very subtle, especially if vision is normal. It is therefore important to ensure a thorough ophthalmic examination with relevant imaging, allowing for a holistic view of the patient's history, examination and investigation results.

In this case, the patient developed asymptomatic subretinal fluid subsequent to the cataract operation. It is important to consider other differentials of macula oedema, particularly postoperative cystoid macular oedema (CMO). Notably, cystoid macular oedema is characterised by a thickened retina on OCT, accompanied by typically intra-retinal fluid displayed in a cystic pattern; neither of these features was observed in this patient, rendering this unlikely as a cause [11].

Central serous chorioretinopathy is an established cause for the abrupt development of subretinal fluid. While its aetiology remains largely unclear, steroid use is considered a potential risk factor; other risk factors, such as alcohol use and uncontrolled hypertension, were not present in this patient [12]. Although the choroid is classically thickened in this condition, as part of the so-called 'pachychoroid' spectrum of diseases, it can also occur with a normal thickness [13]. Given the patient’s medical history, the improbability of alternative causes, and the temporal association with steroid use, CSCR was deemed the most likely diagnosis.

Although not conducted in this case, fluorescein angiography can be useful in differentiating causes of macular oedema. CSCR typically manifests a distinctive 'ink blot' or 'smokestack' pattern, in contrast to the 'petalloid' leakage observed in CMO and varying patterns associated with other causes of macular oedema [13,14].

Despite the development of subretinal fluid, the patient remained asymptomatic with improved visual acuity back to baseline. Therefore, a monitoring approach was used over time, revealing mostly unchanged results. However, at the three-month follow-up for the left eye cataract surgery, following the recent discontinuation of dexamethasone, the findings were spontaneously resolved bilaterally, as is typical for CSCR.

Furthermore, his OCT and retinal changes remained stable over a two-year observation period, leading to the patient’s discharge from further follow-ups with the department.

## Discussion

While Alport syndrome remains a rare condition, occurring in 1 in 50,000 births, it is important to adeptly appreciate and identify its manifestations through various imaging modalities, whilst also excluding other potential differentials [3].

Although existing literature presents some instances of ocular photos and OCT demonstrations related to Alport syndrome, these instances are limited and often involve subtle findings [4,7,8]. In this particular case, the classical findings are more pronounced due to the severity of the patient’s condition, providing a noteworthy example of the disease’s progression.

Moreover, the patient developed central serous chorioretinopathy in response to steroid prophylaxis post-surgery. Given the multitude of causes for the observed subretinal fluid and the patient’s complex medical history, the ophthalmologist must maintain careful consideration of various differentials. Establishing a diagnosis amid such findings can be challenging and requires a comprehensive approach involving a detailed history, clinical examination, investigations and a nuanced understanding of the patient’s overall health. Early diagnosis, facilitated by this thorough approach, contributes to improved patient outcomes.

## Conclusions

In summary, the ability to recognize the findings of Alport syndrome and integrate them with the patient’s complaints and additional clinical signs is crucial for correctly diagnosing and treating this case. Furthermore, documenting and disseminating cases like this one can contribute to the ongoing education of ophthalmologists, offering valuable insights for managing similar patients and enhancing resources related to Alport syndrome.
